# Inspiratory muscle strength and post-liver transplantation outcomes: safety of measurement in the ICU ‒ an observational study

**DOI:** 10.1016/j.clinsp.2026.101079

**Published:** 2026-09-22

**Authors:** Rayssa Pistilli Duarte, Rodrigo Marques Tonella, Ligia dos Santos Roceto Ratti, Lilian Elisabete Bernardes Delazari, Gabriela Maria de Oliveira, Leticia de Souza Campos Assis, Mariana Bueno da Rosa, Edmilson Florêncio Pinto Junior, Enrico de Francisco Magnani, Ilka de Fátima Santana Ferreira Boin

**Affiliations:** a Hospital de Clínicas da Universidade Estadual de Campinas (UNICAMP), Campinas, SP, Brazil; b Department of Physical Therapy, EEFFTO – Universidade Federal de Minas Gerais (UFMG), Belo Horizonte, MG, Brazil; c LabCare Research Laboratory, Universidade Federal de Minas Gerais (UFMG), Belo Horizonte, MG, Brazil; d Faculdade de Ciências Médicas (FCM), Universidade Estadual de Campinas (UNICAMP), Campinas, SP, Brazil

**Keywords:** Liver transplantation, Respiratory muscle strength, Postoperative recovery, Inspiratory pressure

## Abstract

**Objective:**

Assess respiratory muscle strength and hemodynamic responses in the early postoperative period after Liver Transplantation (LT) and to analyze their associations with clinical outcomes.

**Patients and methods:**

A prospective and observational study included adult LT recipients admitted to the Intensive Care Unit (ICU) of the Clinical Hospital of the University of Campinas. Inspiratory muscle strength was assessed during the first three postoperative days using a digital Manovacuometer and *POWERbreathe®* device. Hemodynamic and respiratory parameters were continuously monitored. Statistical analyses included repeated-measures ANOVA, Wilcoxon tests, correlation analyses (p < 0.05), linear mixed models for respiratory variables, and multiple linear regression to examine the directionality of associations.

**Results:**

Sample consisted of 35 patients, with a median age of 59-years [IQR: 52.0–68.0] and a predominance of males (n = 26; 74.3%). *The MELD* mean was 18.1±7.6, SAPS3 score of 44.7±11.2, and SOFA score of 9.2±3.1, MIP increased significantly between postoperative days 1 and 3 (37.5 [32.8–53.8] vs. 49.0 [40.0–72.0] cmH_2_O, p = 0.035). The S-Index showed significant negative correlations with both surgery duration (*r_s* = -0.52, p = 0.009) and ICU length of stay (*r* = -0.42, p = 0.015). No clinically relevant hemodynamic changes occurred during tests; however, there was a main effect of Time for MAP (4.079, 204.0) = 6.765, p < 0.0001) and RR (2.030, 101.5) = 3.628, p = 0.0295).

**Conclusions:**

LT patients exhibit early postoperative improvement in inspiratory muscle strength, with both assessment methods proving safe. Higher dynamic inspiratory strength is associated with shorter ICU stay and surgical duration, supporting the inclusion of routine respiratory muscle monitoring in postoperative physiotherapy protocols.

## Highlights

• Inspiratory muscle strength measurement is safe in liver transplant ICU patients.
• No adverse events were observed during inspiratory strength assessment.
• Lower inspiratory strength is associated with worse postoperative outcomes.
• Inspiratory muscle weakness may identify high-risk patients early.

## Introduction

Liver Transplantation (LT) is the standard treatment for end-stage liver disease and selected hepatic tumors, significantly improving patient survival. However, postoperative complications, particularly respiratory, remain frequent and may adversely affect recovery.^1^, ^2^, ^3^, ^4^, ^5^, ^6^, ^7^

Respiratory muscle weakness has been identified as an important determinant of clinical outcomes, being associated with prolonged mechanical ventilation and disease severity. Therefore, assessment of diaphragmatic function and respiratory muscle strength is essential for monitoring pulmonary performance and guiding rehabilitation strategies throughout the perioperative period.^8^, ^9^, ^10^, ^11^, ^12^, ^13^, ^14^, ^15^

Physiotherapy is essential in this process, using tools such as manovacuometry and electronic devices for dynamic evaluation of inspiratory muscle performance.^16^^,^^17^

Despite advances in rehabilitation, evidence regarding physiotherapeutic assessment in the immediate postoperative period of LT remains limited, highlighting the need for further investigation to optimize early detection of respiratory impairment and improve clinical and functional outcomes.^18^, ^19^, ^20^

## Objective

The aim of this study was to longitudinally evaluate the S-Index and Maximal Inspiratory Pressure (MIP) during the early postoperative period after liver transplantation, investigate their associations with clinical variables and postoperative outcomes, and compare the safety, physiological responses, and hemodynamic effects of measurements obtained using the *POWERbreathe®* device and a digital manovacuometer.

## Materials and methods

### Study design and setting

This was an observational, longitudinal, and prospective study conducted in the Liver Transplantation Intensive Care Unit (LT-ICU) of the Clinical Hospital of the University of Campinas (CH-UNICAMP), Brazil. Participants were followed from admission to the unit until hospital discharge. The sample size was determined by convenience, including all eligible patients admitted during the study period.

### Participants

Adult patients (≥ 18 years) undergoing Liver Transplantation (LT) and admitted to the LT-ICU were eligible for inclusion if they required invasive mechanical ventilation and subsequently underwent successful elective extubation, defined as the absence of the need for mechanical ventilation for more than 48 hours, in accordance with the institutional protocol. Written informed consent was obtained from all participants. Assessments of inspiratory muscle function, using the *POWERbreathe®* device and a digital manovacuometer, were performed after extubation and within the first three days following extubation in the ICU.

Exclusion criteria included preoperative invasive mechanical ventilation, need for retransplantation, inability to complete initial testing, respiratory instability precluding assessment, and impaired consciousness or cognitive status.

Patients who were not included in the study, as well as those excluded during follow-up, remained under the routine care of the institutional liver transplantation program and continued to receive comprehensive postoperative management from the multidisciplinary intensive care unit team according to institutional protocols. Participation in the study was exclusively observational and did not influence clinical decision-making, therapeutic interventions, or patient monitoring. Consequently, exclusion from the research protocol did not result in any restriction of care or loss of access to standard treatment. Clinical outcomes of non-participants and excluded patients were not prospectively collected by the research team, as these individuals were not part of the study cohort.

### Ethical considerations

The study was approved by the Research Ethics Committee of the University of Campinas (UNICAMP), Brazil, in accordance with Resolution n° 466/2012 of the Brazilian National Health Council. The research protocol was approved on January 17, 2020 (CAAE number 22427219.3.0000.5404; approval number 3.802.788). The study was registered in the Brazilian Registry of Clinical Trials (ReBEC) under registration number RBR-3v4k4d6.

All participants or their legal representatives signed a written informed consent form prior to enrollment.

Clinical and demographic data were obtained from medical records and physiotherapy team reports. Variables included age (years), sex (male/female), Model for End-Stage Liver Disease (*Score MELD*), duration of mechanical ventilation (hours), surgical time (hours), and intraoperative blood product volume (milliliters). All information was recorded on a standardized evaluation form.

### Positioning and monitoring

During the assessments, patients were placed in a semi-recumbent position with the head of the bed elevated between 35° and 40°. Continuous monitoring was performed using a multiparameter monitor to record heart rate (beats per minute – bpm), respiratory rate (breaths per minute – bpm), mean arterial pressure (mmHg), and peripheral oxygen saturation (SpO_2_, %).

### Evaluation protocol

After LT-ICU admission, patients were followed by the principal investigator. Data collection began 24-hours after elective orotracheal extubation. Assessments using the *POWERbreathe®* device were performed daily until transfer to the ward, whereas measurements with the digital Manovacuometer were performed on alternate days, with the first assessment conducted on the day the post-extubation evaluation protocol was initiated and the second assessment performed on the third and final day of follow-up.

To minimize the risk of inducing respiratory muscle fatigue in an already vulnerable population, characterized by pre-existing muscle weakness and frequently associated with sarcopenia, MIP was assessed on alternate days, as recommended to reduce the cumulative burden of sustained maximal efforts and their potential fatigue-related effects on respiratory muscles. In contrast, the S-Index was measured daily because, by its nature, it represents a shorter and more dynamic maximal inspiratory effort, requiring less sustained contraction time and being less likely to generate cumulative fatigue when compared to MIP.

The literature indicates that patients with advanced liver disease commonly exhibit reduced respiratory muscle strength and fatigue symptoms related to systemic impairment and sarcopenia, thereby justifying the need for protocols that balance assessment sensitivity with safety when repeating maximal effort measurements. Previous studies have demonstrated that respiratory muscle fatigue may be induced by repeated maximal efforts in fragile populations, supporting the rationale for spacing more demanding tests, while shorter and dynamic measures can be performed more frequently without compromising respiratory muscle integrity.

Not all patients remained in the ICU for three consecutive days; therefore, a progressive reduction in sample size was observed across the assessment days due to ICU discharge or unavailability for continued follow-up. Nevertheless, all patients included in the analysis underwent a minimum of two *S-Index* assessments and one MIP measurement.

Vital signs were continuously monitored and recorded at six time points:

• Pre-test;
• During the test;
• Immediately after completion;
• At 1-minute of rest;
• At 5-minutes of rest, and
• At 10-minutes of rest.

These recordings allowed analysis of the physiological response to effort and recovery to baseline values.

### Dynamic inspiratory muscle strength assessment – POWERbreathe®

Dynamic inspiratory muscle strength was assessed using the *POWERbreathe®* electronic inspiratory muscle training device (model KH2, IMT Technologies Ltd., Birmingham, UK), registered with the Brazilian Health Regulatory Agency (ANVISA) under Medical Device Registration Number 81001390007, connected to a Dell Intel Pentium computer running *Breathelink®* software.

The mouthpiece was positioned between the patient’s lips, with a nasal clip applied. Participants were instructed to perform 10 consecutive deep inspirations without pause through a valve allowing free airflow.

The device recorded inspiratory flow and calculated inspiratory strength (cmH_2_O), generating the *S-Index*, which reflects the relationship between inspiratory pressure and flow.

### Maximal inspiratory pressure assessment – Digital Manovacuometer

Maximal Inspiratory Pressure (MIP) was measured using a portable digital Manovacuometer (MVD 300, Globalmed®, São Paulo – SP ‒ BRAZIL), digital manometer similarly has ANVISA registration with Medical Device Registration Number: 10320099006. The mouthpiece was positioned between the lips with a nasal clip in place.

Participants were instructed to exhale to residual volume, followed by a maximal sustained inspiration. Three consecutive maneuvers were performed, with 30-second intervals between trials and a 120-second rest at the end of the series.

The highest value obtained among the three attempts was recorded for analysis using a standardized form. Measurements were performed on alternate days, depending on the patient’s clinical tolerance.

The assessment of MIP values was performed on alternate days in order to minimize physiological burden on the study population. Given the vulnerability of the sample, which consisted of patients who had undergone major surgery and were in the early postoperative period, longer intervals between measurements were adopted to reduce the risk of influencing the results and to prevent potential respiratory complications associated with repeated effort, such as respiratory muscle fatigue.

### Bias control

Efforts were made to minimize potential sources of bias. Selection bias was reduced by consecutively including all eligible patients during the study period.

To minimize measurement bias, inspiratory muscle strength assessments were performed using a standardized protocol, with patients positioned in a semi-recumbent position (35°–40°) and using the same equipment for all measurements.

In addition, evaluators were previously trained to perform the tests, aiming to reduce interobserver variability.

Potential confounders, including sedation, analgesia, vasoactive drug use, pain, fatigue, and level of cooperation, were considered in the analysis and interpretation of the results.

Despite these measures, residual bias inherent to observational studies cannot be excluded.

### Sample size

The sample size was determined by the number of patients meeting the inclusion criteria during the study period. Due to the exploratory nature of the study and the specific population (liver transplant recipients in the ICU), a convenience sample was used.

### Quality control and standardization of assessments

All respiratory assessments and data collection procedures were performed by the same trained research team throughout the study period. To ensure standardization and reproducibility, the measurements were conducted according to predefined protocols and institutional Standard Operating Procedures (SOPs) for the management of LT recipients and the operation of the assessment devices. Patient safety was prioritized at all times, and evaluations were performed within each participant’s clinical tolerance and physiological limits, with interruption of testing whenever deemed necessary by the clinical or research team.

### Statistical analysis

All statistical analyses were performed using SPSS software (version 25, IBM Corporation) and GraphPad Prism (version 6.01, GraphPad Software, San Diego, CA, USA). The significance level adopted for all analyses was set at 0.05.

Unadjusted and confounder-adjusted estimates are presented with their corresponding 95% Confidence Intervals. Multivariable models were constructed to account for potential confounding factors, including age, sex, duration of mechanical ventilation, and the use of sedation and vasoactive drugs. These variables were selected a priori based on their clinical relevance and previously reported associations with respiratory muscle function.

Initially, continuous variables were assessed for normality using the Shapiro-Wilk test. Variables with a normal distribution were presented as mean ± Standard Deviation (mean ± SD), whereas non-normally distributed variables were presented as median and interquartile range (median [IQR: P25–P75]). Categorical variables were expressed as frequencies and percentages.

Paired comparisons between two time points (Day-1 vs. Day-3) were performed using the Wilcoxon signed-rank test. To evaluate hemodynamic and respiratory variables (heart rate, systolic blood pressure, diastolic blood pressure, mean arterial pressure, respiratory rate, and SpO_2_) across multiple factors, a two-way repeated measures analysis of variance was applied. This analysis included two within-subject factors: “Test” (levels: MIP vs. S-Index) and “Time” (levels: pre, during, immediate post, post 1 min, post 5-min, and post 10-min).

The assumption of sphericity was assessed, and when violated, the Greenhouse-Geisser correction was applied. Sidak-adjusted post hoc tests were used for multiple comparisons, examining both interaction and main effects. To further detail the main effect of Time, separate one-way repeated measures ANOVAs were conducted for the S-Index and MIP tests.

For the longitudinal analysis of respiratory variables (MIP and *S-Index*) across the three assessment time points, Linear Mixed Models (LMMs) were employed. This approach was selected due to its ability to handle unbalanced data resulting from follow-up losses (missing data), using Restricted Maximum Likelihood (REML) estimation. Post hoc multiple comparisons were adjusted using the Sidak method.

Bivariate associations between outcome variables, severity scores, and inspiratory measures were assessed using Pearson (*r*) or Spearman (*r_s_*) correlation coefficients, depending on data distribution. To determine the independent influence of explanatory variables on outcomes, multiple linear regression analyses were conducted, controlling for potential confounding factors (such as surgical duration). Continuous variables were not categorized.

## Results

During the data collection period (May 2023 to August 2025), 84 liver transplants were performed at CH-UNICAMP and admitted to the LT-ICU.

Among these, 13 patients died before elective extubation, 4 of them had no level of consciousness for testing, 4 had been intubated and mechanically ventilated before transplantation, and 10 declined to sign the informed consent form prior to surgery; therefore, 53 eligible participants.

Data collection was not performed in 7 individuals, at certain time points planned in the study protocol due to logistical limitations of the research team. Specifically, transportation difficulties and scheduling constraints that prevented the researchers from traveling to the hospital precluded the execution of assessments during these periods. It is emphasized that these occurrences were operational in nature and were not related to the participants were not related to the clinical condition of the participants, standard care procedures, or methodological flaws of the study, resulting in 46 participants. Of these, patients were excluded due to respiratory instability preventing test performance (n = 4), reintubation after elective extubation (n = 3), or withdrawal of consent (n = 4). Thus, the final sample comprised 35 patients (Fig. 1).

**Fig. 1:**
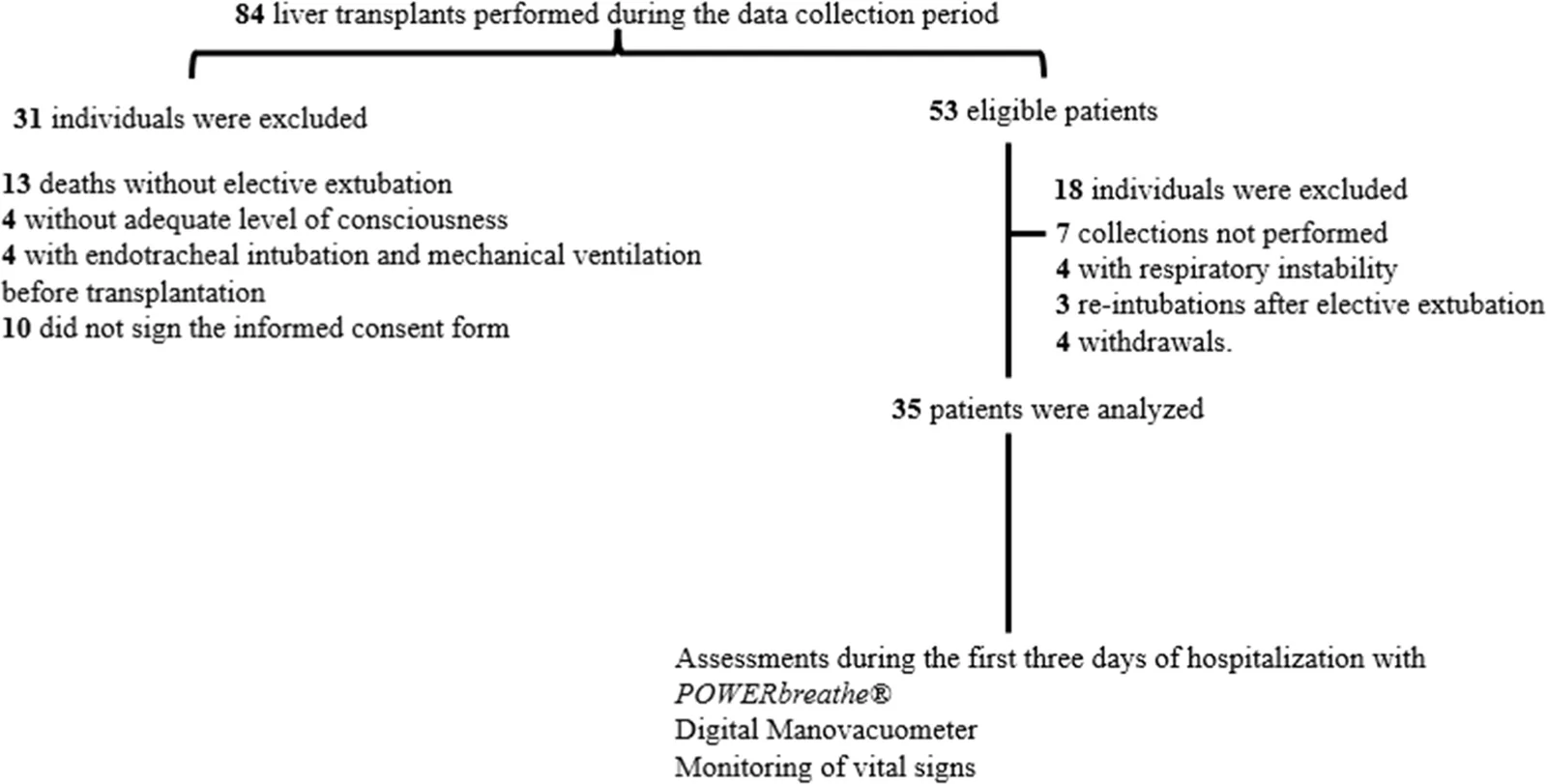
Flowchart of the study population.

The study sample consisted of 35 patients, with a median age of 59-years [IQR: 52.0–68.0] and a predominance of males (n = 26; 74.3%). The median waiting time for transplantation was 255-days [IQR: 98–382]. Regarding severity scores, the sample presented a mean *MELD*-Na score of 18.1±7.6, a mean SAPS3 score of 44.7±11.2, and a mean SOFA score of 9.2±3.1. According to the review of medical records, none of the participants had a documented diagnosis of pulmonary disease in the medical records that could potentially confound the assessment of inspiratory muscle strength measured using the *POWERbreathe®* device and digital Manovacuometer.

Detailed sociodemographic, anthropometric, and clinical characteristics of the sample are shown in Table 1.

**Table 1:** Sociodemographic, anthropometric, and clinical characteristics of the sample.

Variable	Value
Sociodemographic variables	
Age (years)	59 [52‒68]
Sex, n (%)	
Female	9 (25.7%)
Male	26 (74.3%)
Anthropometric variables	
Weight (kg), mean ± SD	71.97 ± 13.78
Height (cm), mean ± SD	167.90 ± 7.23
Clinical variables and severity scores	
Waiting time for transplantation (days), median [IQR]	255 [98‒382]
Score MELD-Na, mean ± SD	18.11 ± 7.62
SAPS3 score, mean ± SD	44.66 ± 11.15
SOFA score, mean ± SD	9.22 ± 3.12

Data are presented as mean ± Standard Deviation (SD); median [interquartile range (IQR)] or absolute (n) and relative (%) frequencies. %, Percent; kg, Kilogram; cm, Centimeter; Score MELD-Na, Model for End-Stage Liver Disease incorporating Sodium; SAPS3, Simplified Acute Physiology Score III; SOFA, Sequential Organ Failure Assessment.

The median surgical time was 300 minutes [IQR: 255–360]. Regarding hospitalization outcomes, patients remained in the ICU for a median of 5-days [IQR: 4–8] and had a total hospital stay of 10-days [IQR: 8–15]. Additional data related to intraoperative variables and clinical outcomes are presented in Table 2.

**Table 2:** Surgical procedure variables and clinical outcomes.

Variable	Value
Ischemia times	
Warm ischemia (min), mean ± SD	46.14 ± 10.34
Cold ischemia (min), mean ± SD	369.70 ± 103.80
Intraoperative variables	
Surgical time (min), median [IQR]	300 [255‒360]
Blood loss (mL), median [IQR]	1200 [1000‒2000]
Postoperative Outcomes	
Total mechanical ventilation time (SC + ICU, min), median [IQR]	740 [555‒1373]
Mechanical ventilation time (ICU, min), median [IQR]	525 [252,5‒1069]
ICU stay (days), median [IQR]	5 [4‒8]
Total hospital stay (days), median [IQR]	10 [8 15]

Data are expressed as mean ± SD or median [IQR]. min, Minutes; mL, Milliliter; MV, Mechanical Ventilation; OR, Operating Room; ICU, Intensive Care Unit.

Baseline values of respiratory pressures (*S-Index* and MIP) were assessed during the first three postoperative days, as shown in Table 3. Mean *S-Index* values (normally distributed) remained stable across the three days, being 28.2±11.3 cmH_2_O on Day-1 (D1), 30.5±12.0 cmH_2_O on Day-2 (D2), and 30.2±10.6 cmH_2_O on Day-3 (D3). For Maximal Inspiratory Pressure (MIP, non-normally distributed), the median values were 37.5 cmH_2_O [IQR: 32.8–53.8] on D1 and 49.0 cmH_2_O [IQR: 40.0–72.0] on D3.

**Table 3:** Respiratory pressure values – *S-Index* (Days-1 to 3) and MIP (Days-1 and 3).

Variable (n)	Value	p-value
*S-Index* (cmH_2_O)		0.030
Day-1 (35)	28.21 ± 11.25	
Day-2 (35)	30.47 ± 11.95	
Day-3 (21)	30.20 ± 10.56	
MIP (cmH_2_O)		0.035
Day-1 (28)	37.50 [32.75–53.75]	
Day-3 (19)	49.00 [40.00–72.00]	

Data are presented as mean ± Standard Deviation (SD) or median [Interquartile Range (IQR)]. n, Number of participants; cmH_2_O, Centimeters of water; MIP, Maximum Inspiratory Pressure.

A progressive reduction in sample size was observed over the follow-up days, primarily due to discharges from the Intensive Care Unit (ICU). As patients showed clinical improvement and hemodynamic stability, they were transferred to lower-acuity care units, which precluded the continuation of subsequent assessments planned in the study protocol. Therefore, the decrease in the number of participants at later assessment time points was not related to losses due to exclusion, death, or adverse events, but rather reflected the favorable clinical course of the studied population.

A Wilcoxon signed-rank test was performed to investigate whether there was a difference in MIP values between first assessment (D1) and the second assessment (D3). The median MIP on D1 was 38.0 cmH_2_O [IQR: 35.0–55.0], and on D3 it was 49.5 cmH_2_O [IQR: 40.0–72.75]. The median difference between D3 and D1 was 5.5 cmH_2_O.

The results demonstrated a statistically significant difference between MIP values at the two time points (p = 0.035) (Fig. 2).

**Fig. 2:**
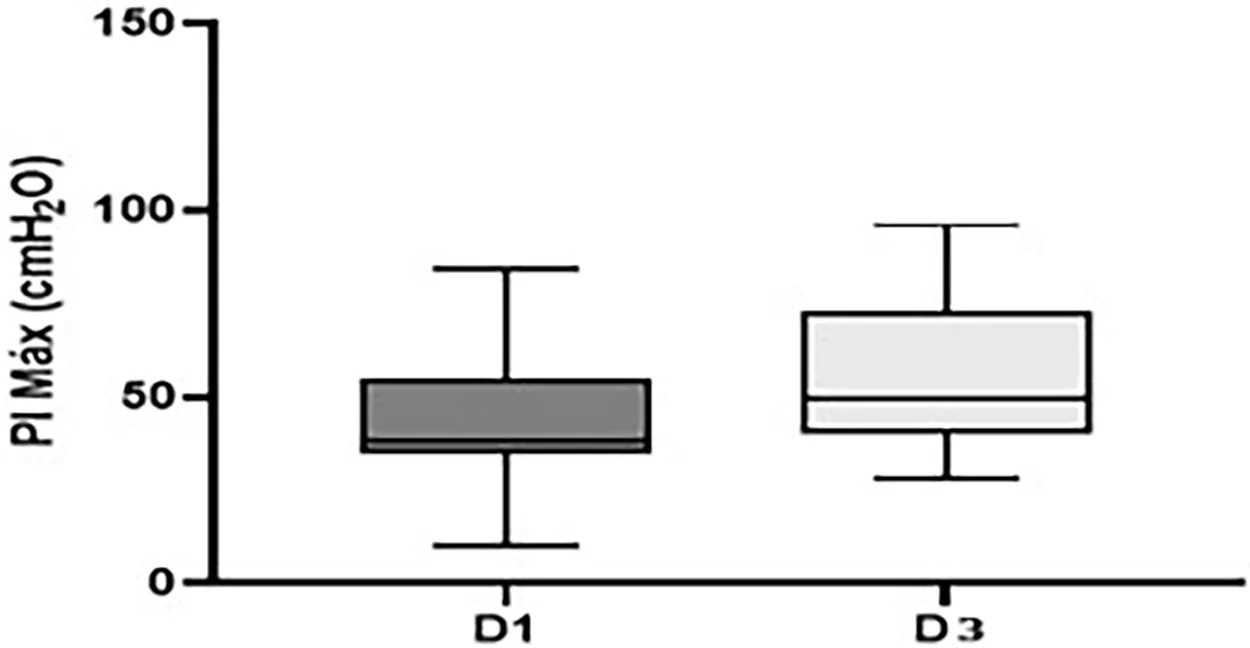
Median values of Maximal Inspiratory Pressure (MIP) on postoperative Days-1 (D1) and 2 (D3).

Another analysis of *S-Index* progression was conducted using a Linear Mixed Model (LMM) with a random intercept to accommodate the unbalanced data structure resulting from follow-up losses (missing data) due to hospital discharge. Multiple comparisons adjusted by the Sidak method revealed a statistically significant increase in *S-Index* values between Day-1 (mean = 26.01; SD = 10.62) and Day-3 (mean = 30.20; SD = 10.56; Δ = 3.87±1.07; p = 0.003). Again, no significant differences were observed between Day-1 and Day-2 (mean = 28.95; SD = 12.07; p = 0.299) or between Day-2 and Day-3 (p = 0.494), corroborating the repeated-measures ANOVA results and suggesting that recovery of this parameter occurs more markedly over a longer time interval (Fig. 3).

**Fig. 3:**
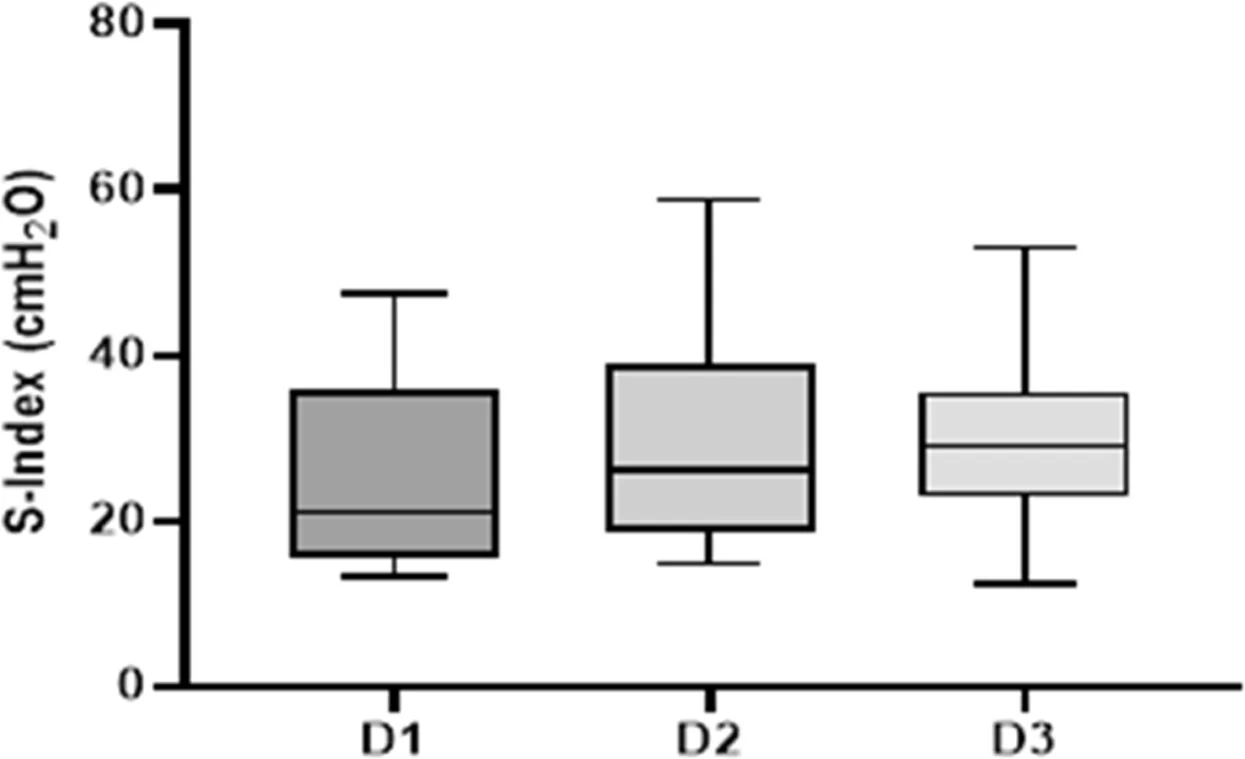
Mean *S-Index* values on postoperative Days-1 (D1), 2 (D2), and 3 (D3).

To evaluate hemodynamic and respiratory responses, a two-way repeated measures ANOVA was conducted with two within-subject factors: Test (MIP vs*. S-Index*) and Time (six time points: pre, during, immediately post, 1-min post, 5-min post, and 10-min post).

This analysis revealed no statistically significant Test × Time interaction for any of the variables assessed: HR (5,250) = 0.7945, p = 0.554), SBP (5,250) = 0.7105, p = 0.6161), DBP (5,250) = 0.9762, p=0.4328), MAP (5,250) = 1.474, p = 0.1988), RR (5,250) = 0.8007, p = 0.5500), and SpO_2_ (5,250) = 0.4682, p = 0.7998).

This indicates that the response patterns over the six times were similar between the MIP and *S-Index* tests. Likewise, no main effect of Test was found for any variable (HR: p = 0.6545; SBP: p = 0.8644; DBP: p = 0.9205; MAP: p = 0.8549; RR: p = 0.8140; SpO_2_: p = 0.6128).

Multiple comparisons confirmed no significant differences between MIP and *S-Index* at any of the time points.

However, there was a main effect of Time for MAP (4.079, 204.0) = 6.765, p < 0.0001) and RR (2.030, 101.5) = 3.628, p = 0.0295), indicating that these variables changed significantly across the time points, regardless of the test performed. No main effect of Time was found for HR (p = 0.0928), DBP (p = 0.1406), or SpO_2_ (p = 0.2469).

To further explore the significant main effect of Time, repeated-measures analyses were conducted separately for the *S-Index* and MIP tests on the MAP and RR variables. The hemodynamic and respiratory data are presented in Table 4.

**Table 4:** Hemodynamic and respiratory responses across six assessment moments during the *S-Index* test and MIP test on D1 of the collection.

Variable	Pre	During	IM-Post	1-min Post	5-min Post	10-min Post	p-value
*S-Index*							
HR (bpm)	88 ± 10	89 ± 10	89 ± 11	88 ± 12	87 ± 10	88 ± 11	0.0928
SBP (mmHg)	130 ± 19	133 ± 20	133 ± 20	132 ± 18	130 ± 19	128 ± 19	0.0290
DBP (mmHg)	66 ± 10	68 ± 10	66 ± 9	67 ± 11	66 ± 9	65 ± 9	0.1406
MAP (mmHg)	87±10^a^	91±11^a^	89 ± 9^a^	88 ± 11	86 ± 10	85 ± 9^a^	0.0003
RR (bpm)	20 ± 6	21 ± 7	24 ± 7^a^	22 ± 8	22 ± 7	19 ± 5^a^	0.0078
SpO_2_ (%)	95 ± 3	95 ± 3	94 ± 3	94 ± 3	95 ± 2	95 ± 2	0.2469
MIP							
HR (bpm)	88 ± 11	90 ± 12	91 ± 13	90 ± 12	89 ± 12	88 ± 11	0.0928
SBP (mmHg)	130 ± 21	133 ± 20	132 ± 19	129 ± 18	129 ± 22	128 ± 19	0.1028
DBP (mmHg)	66 ± 8	67 ± 10	68 ± 10	66 ± 11	67 ± 9	66 ± 9	0.1406
MAP (mmHg)	87 ± 10	89 ± 11	89 ± 11	85 ± 10	86 ± 11	87 ± 11	0.0708
RR (bpm)	20 ± 6	23 ± 8^a^	23±7^a^	21±7	21±6	19±5^a^	0.0004
SpO_2_ (%)	95 ± 2	95 ± 2	94 ± 7	95 ± 3	95 ± 3	95 ± 2	0.2469

Data are presented as Mean ± Standard Deviation (SD), rounded to the nearest integer; p-value ‒ p<0.05 for comparisons detailed in the text. IM-pos, Immediate pos; HR, Heart rate; SBP, Systolic Blood Pressure; DBP, Diastolic Blood Pressure; MAP, Mean Arterial Pressure; RR, Respiratory Rate; SpO_2_, Peripheral Oxygen Saturation; bpm, Beats per minute; mmHg, Millimeters of Mercury; %, Percent.

a Statistically significant**.**

For MAP, a repeated-measures ANOVA with Geisser-Greenhouse correction was applied. In the *S-Index* test, a statistically significant effect of time was observed (3.672, 91.79) = 6.200, p = 0.0003, ε = 0.7343). Tukey’s post hoc test indicated that MAP was higher during the During phase (M = 90.77) compared with Pre (M = 86.50; p = 0.0355) and Post 10-min (M = 84.69; p = 0.0045). The Immediate Post phase (M = 89.04) was also significantly higher than Post 10-min (p = 0.0106). In contrast, in the MIP test, no significant effect of time was found (3.585, 89.62) = 2.308, p = 0.0708, ε = 0.7170).

For RR, which did not show a normal distribution, the Friedman test was used. Both tests showed a statistically significant effect of time. In the *S-Index* test (χ^2^(5) = 16.70, p = 0.0051), Dunn’s post hoc test revealed that RR at the Immediate Post time point was significantly higher than at Post 10-min (p = 0.0023). In the MIP test (χ^2^(5) = 22.51, p = 0.0004), Dunn’s post hoc test indicated that RR was higher during the During (p = 0.0112) and Immediate Post (p = 0.0027) time points compared with Post 10-min.

However, despite these statistically significant findings, the magnitude of the changes remained within normal physiological ranges, indicating the safety of using the device.

To investigate the association between respiratory pressures, severity scores, and clinical outcomes, Pearson’s or Spearman’s correlation coefficients were calculated according to data distribution.

A positive and statistically significant Spearman correlation was observed between hospital length of stay and the severity scores SAPS 3 (*r_s_* = 0.5378, p=0.0018), *Score MELD*-Na (*r_s_* = 0.3890, p=0.0230), and CHILD (*r_s_* = 0.4027, p=0.0201).

Conversely, the *S-Index* showed significant negative correlations with both surgery duration (*r_s_* = -0.52, p=0.009) and ICU length of stay (*r* = -0.42, p=0.015) (Fig. 4). These findings suggest that greater dynamic inspiratory strength is associated with shorter ICU stays and reduced surgical time.

**Fig. 4:**
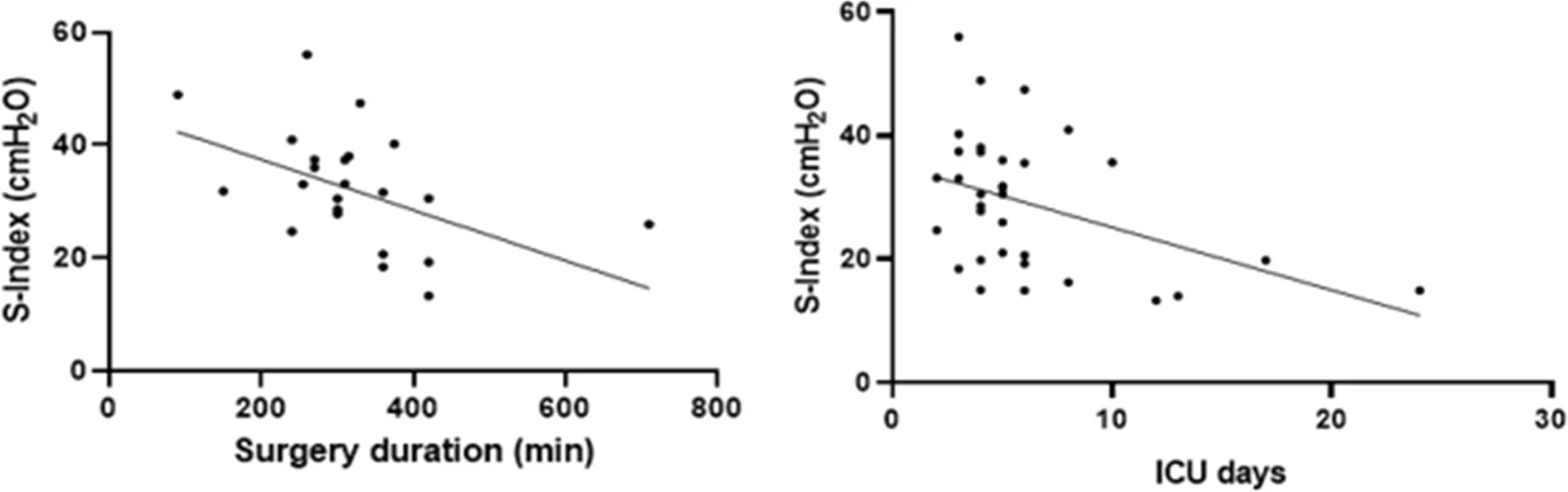
Correlation between *S-Index* values and both surgery duration and ICU length of stay.

To examine the directionality of these associations, a multiple linear regression analysis was performed using *S-Index* and surgical duration as predictor variables and ICU length of stay as the dependent variable. This analysis demonstrated that *S-Index* is a significant predictor of ICU length of stay, independent of surgical duration (p = 0.024). For each one-unit increase in *S-Index*, an estimated reduction of 0.67-days in ICU stay was observed (B = -0.67; 95% CI: -1.24 to -0.09). This inverse association was corroborated by a first-order partial correlation analysis, which revealed a moderate negative association between *S-Index* and ICU days (*r_partial* = -0.399; p = 0.024) after controlling for surgical duration.

No additional analyses, such as subgroup, interaction, or sensitivity analyses, were performed, given the exploratory nature and limited sample size of the study.

## Discussion

The present study evaluated respiratory muscle function in patients undergoing liver transplantation, focusing on the evolution of inspiratory strength indices (MIP and *S-Index*) during the early postoperative period and their relationship with clinical and hemodynamic variables. The sample was predominantly composed of male individuals with a median age of 59-years, a profile consistent with previous reports of liver transplant populations, which describe a higher prevalence of male recipients in their sixth decade of life.^21^, ^22^, ^23^

The significant improvement observed in MIP between the first and second postoperative days suggests an early recovery of inspiratory strength. This improvement may reflect the initial hemodynamic and metabolic stabilization following surgery, as well as the reduction of anesthetic effects and pre-existing respiratory muscle fatigue.^24^^,^^25^ Previous studies have highlighted that impairment of respiratory muscle strength is common among liver transplant candidates due to sarcopenia and hepatic myopathy ‒ conditions frequently associated with advanced cirrhosis.^26^^,^^27^ Therefore, the increase in MIP within the first 48-hours may indicate a potential for early functional recovery resulting from the restoration of hepatic function and improved tissue perfusion.^28^

The findings regarding the *S-Index* showed a significant increase only between D1 and D3, indicating that recovery of dynamic inspiratory strength occurs more gradually. This pattern aligns with the observations of van den Berg-Emons et al.^20^ and Dronkers et al.,^29^ who reported a progressive improvement in overall muscle function during the first postoperative week as patients regain full spontaneous ventilation and respiratory motor control. The absence of differences between D2 and D3 may be related to individual variability and the residual effects of sedation, which are still common during the early postoperative period.^30^

The absence of significant differences in hemodynamic and respiratory responses between the MIP and *S-Index* tests demonstrates the clinical safety of both methods in this population. This finding is consistent with recent evidence showing that respiratory strength assessments are well tolerated even in critically ill patients when conducted in a standardized and well-monitored manner.^31^^,^^32^ The transient increase in Mean Arterial Pressure (MAP) and Respiratory Rate (RR) during and immediately after the tests likely reflects momentary autonomic activation induced by inspiratory effort, without clinically relevant repercussions.^33^

The negative correlations observed between the *S-Index* and both ICU length of stay and surgical duration suggest that patients with greater inspiratory muscle strength experienced a more favorable postoperative course. These findings reinforce the relevance of respiratory muscle function as a clinically meaningful predictor in the perioperative management of liver transplantation.^34^^,^^35^ Previous studies have consistently demonstrated that respiratory muscle weakness is associated with prolonged mechanical ventilation, a higher incidence of pulmonary complications, and extended hospitalization periods. Such impairments may compromise effective ventilation, cough efficiency, and overall respiratory mechanics, thereby delaying postoperative recovery.^36^^,^^37^

The longitudinal analysis using a linear mixed model demonstrated a significant increase in *S-Index* values between Day-1 and Day-3, indicating a progressive recovery of inspiratory muscle performance in the early postoperative period. The absence of significant differences between adjacent time points suggests that improvements in inspiratory strength may not occur immediately but become more evident over a longer interval. This recovery pattern is consistent with the physiological trajectory observed after major surgery, in which respiratory muscle function is initially impaired by surgical stress, anesthesia, and mechanical ventilation, particularly in critically ill patients. The concordance between the linear mixed model and repeated-measures ANOVA findings strengthens the robustness of these results and supports the use of the *S-Index* as a sensitive marker for monitoring postoperative respiratory muscle recovery.^38^

A multiple linear regression analysis demonstrated that the *S-Index* was an independent predictor of ICU length of stay, even after adjustment for surgical duration, reinforcing the clinical relevance of inspiratory muscle performance in the postoperative period. The observed reduction in ICU stay associated with higher *S-Index* values suggests that patients with better inspiratory strength may achieve earlier clinical stabilization, potentially due to improved ventilatory efficiency and reduced respiratory workload. Respiratory muscle weakness has been widely associated with prolonged mechanical ventilation and delayed recovery in critically ill patients, supporting the biological plausibility of this finding. The consistency between the regression model and partial correlation analysis further strengthens the robustness of the observed association and highlights the *S-Index* as a potentially useful marker for identifying patients at risk for prolonged ICU stay.^39^

From a clinical perspective, while surgical duration is not directly modifiable, its association with inspiratory muscle dysfunction underscores its relevance as a clinical risk marker. This recognition may facilitate early identification of high-risk patients and promote the timely adoption of targeted postoperative respiratory monitoring and rehabilitation strategies, potentially improving recovery.

Although the multiple linear regression analysis identified lower S-Index values as an independent predictor of longer ICU length of stay, the directionality of this association should be interpreted with caution. It is plausible that more complex and prolonged surgical procedures may contribute to increased systemic inflammation, greater metabolic stress, and subsequent postoperative muscle weakness, thereby negatively impacting inspiratory muscle performance. Conversely, reduced preoperative or early postoperative inspiratory muscle strength may predispose patients to a more complicated recovery course. These mechanisms may be mutually reinforcing, and further prospective studies are warranted to better elucidate the causal pathways underlying these associations.

In this context, the *S-Index* emerges as a possible valuable and sensitive parameter for assessing inspiratory muscle performance, particularly because it reflects dynamic inspiratory effort and may capture functional deficits not fully detected by static measurements alone. Early monitoring of inspiratory muscle strength may therefore contribute to improved risk stratification, allowing clinicians to identify patients at greater risk of adverse respiratory outcomes. Furthermore, these assessments may support the implementation of individualized physiotherapeutic interventions, including targeted inspiratory muscle training, with the potential to optimize respiratory function and improve postoperative recovery in liver transplant recipients.

The positive correlations between disease severity scores (SAPS3, *Score MELD*-Na and Child-Pugh) and hospital length of stay confirm the prognostic value of these indices in postoperative outcomes. These results are consistent with the findings of Kim et al.^22^ and Dababneh and Mous^1^ who emphasized the predictive value of *Score MELD*-Na and SAPS3 for morbidity and mortality among liver transplant recipients.

In this study, inspiratory muscle strength was not investigated as an independent prognostic marker, but rather as a functional parameter potentially associated with perioperative characteristics and postoperative outcomes. Given the multifactorial nature of liver transplantation outcomes, the observed associations should be interpreted as exploratory findings and not as evidence of a predictive relationship. Therefore, inspiratory muscle strength was analyzed as a clinical variable that may influence or be associated with postoperative recovery in liver transplant recipients.

Finally, the present results underscore the importance of an integrated approach that combines systematic assessment of inspiratory strength with the early implementation of respiratory rehabilitation programs in the postoperative management of liver transplant patients. Growing evidence suggests that inspiratory muscle training can accelerate functional recovery, reduce mechanical ventilation time, and optimize overall clinical outcomes.^1^^,^^40^ Therefore, the measurement of MIP and *S-Index* may be incorporated into routine clinical practice as simple, safe, and sensitive tools for monitoring the clinical progression of liver transplant recipients.

This study has several limitations that should be considered when interpreting the findings. First, the relatively small sample size may limit statistical power and increase the risk of type II error, potentially underestimating true associations; as a single-center study, the findings may be subject to limited external validity, restricting their generalizability to other settings and patient populations. In addition, the heterogeneity of the studied population and clinical factors such as sedation, analgesia, use of vasoactive drugs, pain, fatigue, and level of cooperation during assessments may have introduced measurement variability, potentially leading to imprecision in the estimates. These factors may have influenced the magnitude of the observed effects and, in some cases, may have attenuated true associations.

Finally, as an observational study, residual confounding cannot be excluded, despite efforts to account for relevant clinical variables.

The findings of this study should be interpreted with caution. While the results suggest that inspiratory muscle strength is reduced in the early post-extubation period following liver transplantation and may be influenced by clinical and perioperative factors, these observations must be considered in light of the study’s limitations, including the small sample size and single-center design.

In addition, the exploratory nature of the analyses and the potential influence of unmeasured confounders may have affected the observed associations. Nevertheless, the results are consistent with previous evidence suggesting that respiratory muscle dysfunction is common in critically ill patients and may impact early postoperative recovery.

Therefore, these findings provide preliminary insights but should be confirmed in larger, multicenter studies with more robust designs. These findings are likely applicable to similar clinical settings but should be extrapolated with caution to different populations and healthcare systems.

## Conclusion

In liver transplant recipients, inspiratory muscle strength, assessed by both MIP and S-Index, improved during the early postoperative period. Higher inspiratory muscle strength was associated with favorable clinical outcomes, including shorter surgical duration and reduced ICU length of stay. Furthermore, assessments performed using the POWERbreathe® device and digital manovacuometer were safe, well tolerated, and not associated with clinically significant hemodynamic alterations. These findings support the feasibility of monitoring inspiratory muscle strength during the early postoperative period after liver transplantation.

## Authors’ contributions

Rayssa Pistilli Duarte: Conceptualization; methodology, validation; investigation; resources; data curation; writing-original draft; writing-review & editing; visualization; supervision; project administration.

Rodrigo Marques Tonella: Data curation; writing-review & editing; visualization; supervision.

Ligia dos Santos Roceto Ratti: Data curation; writing-review & editing; visualization; supervision.

Lilian Elisabete Bernardes Delazari: Formal analysis.

Gabriela Maria de Oliveira: Investigation; resources; data curation.

Leticia de Souza Campos Assis: Investigation; resources; data curation

Mariana Bueno da Rosa: Investigation; resources; data curation.

Edmilson Florêncio Pinto Junior: Investigation; resources; data curation.

Enrico de Francisco Magnani: Formal analysis, writing-review & editing.

Ilka de Fátima Santana Ferreira Boin: Methodology; project administration; visualization; supervision.

## Funding

This study received no external funding, and the authors declare that no financial support was received for the research, authorship, and/or publication of this article.

The data that support the findings of this study are available from the corresponding author upon reasonable request.

## Data availability

The datasets generated and/or analyzed during the current study are available from the corresponding author upon reasonable request.

## Declaration of competing interest

The authors declare no conflicts of interest.
