# Supplementary material for: Inspiratory muscle strength and post-liver transplantation outcomes: safety of measurement in the ICU ‒ an observational study
Source: Clinics (Sao Paulo). 2026 Sep 22;81:101079. doi: 10.1016/j.clinsp.2026.101079 (PMC13629311; doi:10.1016/j.clinsp.2026.101079)
Supplement: Supplementary file 1 [file mmc2.pdf]

PARECER CONSUBSTANCIADO DO CEP

DADOS DO PROJETO DE PESQUISA

Título da Pesquisa: AVALIAÇÃO DA FUNÇÃO DIAFRAGMÁTICA EM PACIENTES SUBMETIDOS A TRANSPLANTE DE FÍGADO EM UNIDADE DE TERAPIA INTENSIVA

Pesquisador: Rayssa Pistilli Duarte

Área Temática:

Versão: 3

CAAE: 22427219.3.0000.5404

Instituição Proponente: Hospital de Clínicas da UNICAMP

Patrocinador Principal: Financiamento Próprio

DADOS DO PARECER

Número do Parecer: 3.802.788

Apresentação do Projeto:

As informações contidas nos campos "Apresentação do Projeto", "Objetivo da Pesquisa" e "Avaliação dos Riscos e Benefícios" foram obtidas dos documentos apresentados para apreciação ética e das informações inseridas pelo Pesquisador Responsável do estudo na Plataforma Brasil.O fígado é a maior glândula do corpo, ocupa inferiormente toda a cúpula diafragmática direita e uma porção da cúpula esquerda, sendo um órgão com múltiplas funções. As células hepáticas ou hepatócitos são células funcionais com papel importante nas funções metabólicas, secretoras e endócrinas, importantes na manutenção da homeostase (WANG e col., 2017; JUZA; PAULI, 2014).Quando disfunções hepáticas se manifestam, consequentemente podem levar à insuficiência hepática, com necessidade de tratamento clínico ou cirúrgico, destacando o transplante hepático (TxH) (CUSTÓDIO e col., 2016).

As disfunções hepáticas que geralmente são indicativas de transplante, de fígado são hepatites dos tipos B,C crônica ou autoimune, doença hepática alcoólica, cirrose biliar primária e colangite esclerosante (PEREIRA e col., 2019). A seleção de candidatos ao transplante hepático (TxH) atualmente é baseado no escore do Model for End-Stage Liver Disease (MELD), independentemente do tempo da lista de espera(PEREIRA e col., 2019).Seu cálculo é feito através dos resultados dos exames de bilirrubina, Razão Normalizada Internacional (RNI)e creatinina. E escores variam de 6 (menor severidade) a 40 (maior severidade), tendo prioridade de alocação do paciente para a
realização do procedimento de substituição do órgão acometido. (GROGAN, 2011; CUSTÓDIO e

col.,
2016).Previamente ao TxH, os pacientes cursam com redução da massa muscular e força, baixa densidade mineral óssea, aumento dos níveis de fadiga e diminuição da capacidade aeróbica, devido disfunção da gliconeogênese e armazenamento de glicogênio, havendo quebra de proteína muscular para uso energético e resultando em perda de peso e fraqueza muscular (DA SILVA e col., 2014).
Outra complicação importante é a ascite, que causa aumento da pressão intra-abdominal e consequentemente aumento da pressão intratorácica, reduzindo a complacência pulmonar, predispondo à atelectasias e derrames pleurais, prejudicando as trocas gasosas (DA SILVA e col., 2012). No entanto, após TxH, fatores como anestesia, trauma cirúrgico, drenagem e uso de cateteres, associados a dor e dificuldade para tossir podem ser observadas e que podem causar complicações da mecânica ventilatória (DA SILVA e col., 2012; DA SILVA e col., 2016). Entre as complicações respiratórias pós-operatórias destaca-se, o derrame pleural, atelectasia, pneumonia, pneumotórax, insuficiência respiratória aguda (IRpA), uso de ventilação mecânica prolongada e o risco de infecções (AYDIN e col., 2015; LUI e col., 2018). Os pacientes transplantados são admitidos em Unidade de Terapia Intensiva (UTI) ventilando mecanicamente através de tubo orotraqueal e uma vez que o suporte ventilatório é instalado, inicia-se um processo imediato de perda de função diafragmática, por desuso, com alterações da performance muscular, redução da força muscular e endurance muscular respiratório. (TONELLA e col., 2017). Além disso, fraqueza muscular respiratória prévia e MELD estão relacionados ao tempo de ventilação mecânica (DUARTE e col., 2018).Diante disso, alguns recursos para avaliação diafragmática, são importantes na análise dessas alterações,bem como acompanhar sua atividade e desempenho desde o período pré-operatório até alta hospitalar(DOS REIS e col., 2019; CUSTÓDIO e col., 2016).
A eletromiografia de superfície (EMGs) é um recurso diagnóstico não invasivo, através de eletrodos na superfície da pele e posicionado no ventre muscular, entre o ponto motor e o tendão distal do músculo. Verifica-se o registro gráfico da atividade elétrica muscular, através dos potenciais de ação, determinada por sinais em microvolts (V) em função do tempo e representados pela raiz quadrática média (RMS) (DA SILVA e col., 2014).De acordo com os estudos de Da Silva e col., 2014 e Limongi, 2014, indivíduos mais comprometidos pela doença hepática apresentaram valores de RMS de diafragma maiores, e podem ser explicados pela necessidade de maior recrutamento muscular para vencer a resistência imposta, gerando maior atividade elétrica. Contudo, é válido

utilizar métodos coadjuvantes a EMGs, capazes de mensurar a força muscular inspiratória, através da avaliação da pressão inspiratória estática máxima (PImáx) ou medida isométrica dos músculos inspiratórios, por meio da medida da pressão negativa gerada na boca durante uma inspiração máxima contra uma via aérea ocluída, logo após de uma expiração forçada próxima ao volume residual (ONAGA e col., 2017; GRAMS e col., 2019; DA SILVA e col., 2012; SILVA e col., 2016).As tentativas devem ser conduzidas com intervalo de um minuto de repouso e devem ser registradas, ao menos, três manobras com variabilidade menor do que 20%, considerando o maior valor obtido (SILVA e col., 2016). Outro recurso, mais recente para avaliação muscular diafragmática, é o dispositivo eletrônico com princípio de treinamento e avaliação da função muscular inspiratória, chamado Powerbreathe®, que diferente da PImáx, é capaz de mensurar a força dinâmica dos músculos inspiratórios, ou seja, em toda amplitude de movimento do tórax durante a inspiração, desde o volume residual até a capacidade pulmonar total (TONELLA e col., 2017; SILVA e col., 2018).

O indivíduo realiza uma manobra de inspiração através de uma válvula de passagem livre de ar onde sensores registram a variação do fluxo inspiratório e a pressão é plotada a cada volume pulmonar, criando uma linha ao longo do tempo, e o ponto mais alto nesta linha é chamado de S-Index (SILVA e col., 2018).E diferentemente da PImáx, quase não há carga imposta (apenas 3 cmH2O) aos músculos inspiratórios e o dispositivo detecta o fluxo de através da válvula e através de algoritmo patenteado é calculada a pressão muscular inspiratória dinâmica (SILVA e col., 2018). Portanto, tendo em vista que os pacientes submetidos ao TxH previamente podem cursar com sarcopenia devido ao mau funcionamento hepático, complicações pós-operatórias e durante a internação na UTI. Por isso, verifica-se a importância de utilizar diferentes métodos de avaliação, com objetivo de detectar possíveis alterações respiratórias e auxiliar em intervenções fisioterápicas adequadas e precoces, a fim de promover melhores desfechos pós-operatórios.Estudo prospectivo e randomizado a ser realizado no Hospital de Clínicas da Universidade Estadual de Campinas durante o segundo semestre de 2019, com a aprovação prévia do comitê de ética através da resolução CNS nº 466. Serão selecionados pacientes que foram submetidos ao procedimento de transplante hepático e após vinte e quatro horas de extubação orotraqueal, cujo familiar ou o próprio participante, quando em condições, ter assinado o Termo de consentimento Livre e Esclarecido (TCLE). Serão incluídos pacientes com idade superior a 18 anos, de ambos os sexos que realizaram a cirurgia de transplante hepático, e que estão extubados a um período mínimo de vinte e quatro horas,

Endereço: Rua Tessália Vieira de Camargo, 126
Bairro: Barão Geraldo **CEP:** 13.083-887
UF: SP **Município:** CAMPINAS
Telefone: (19)3521-8936 **Fax:** (19)3521-7187 **E-mail:** cep@fcm.unicamp.br

estando conscientes e orientados. Serão excluídos do estudo aqueles que não apresentarem nível de consciência ou não compreensão, com necessidade de retransplante, portadores de obesidade grau 3 (IMC maior do que 40,0 Kg/m2) e óbito. Os pacientes incluídos no estudo serão avaliados após 24 horas de 13.083 extubação orotraqueal, com reavaliações diárias durante todo o período de internação na UTI até a alta da UTI para a enfermaria, onde será realizada uma última avaliação. Durante o estudo serão utilizados os seguintes recursos: eletromiografia de superfície (EMGs), dispositivo eletrônico de treinamento muscular inspiratório Powerbreathe® e Manovacuômetro digital. Previamente à avaliação muscular diafragmática, serão registrados em ficha de avaliação informações relacionados ao paciente, procedimento cirúrgico e relacionadas à internação na UTI. Para registros da atividade elétrica muscular diafragmática, será utilizado o aparelho de eletromiografia de superfície da marca EMG System do Brasil (modelo SA1000V3) que estará conectado através de software próprio instalado em um notebook da marca DELL, modelo InterPentiun. A aquisição dos sinais serão feitas em uma frequência de 500Hz e a sensibilidade de amplitude do sinal de 500 VA. Para sua realização o paciente deverá está posicionado em decúbito dorsal, com cabeceira elevada a 35 graus. Serão posicionados quatro eletrodos bipolares auto-adesivos da marca 3M do Brasil®, sendo dois na região paraxifóide, a 5cm de distância, dois na região da margem costal bilateral, à uma margem de 16cm de Estudo prospectivo e randomizado a ser realizado no Hospital de Clínicas da Universidade Estadual de Campinas durante o segundo semestre de 2019, com a aprovação prévia do comitê de ética através da resolução CNS nº 466. Serão selecionados pacientes que foram submetidos ao procedimento de transplante hepático e após vinte e quatro horas de extubação orotraqueal, cujo familiar ou
o próprio participante, quando em condições, ter assinado o Termo de consentimento Livre e Esclarecido (TCLE).Serão incluídos pacientes com idade superior a 18 anos, de ambos os sexos que realizaram a cirurgia de transplante hepático, e que estão extubados a um período mínimo de vinte e quatro horas, estando conscientes e orientados.Serão excluídos do estudo aqueles que não apresentarem nível de consciência ou não compreensão, com necessidade de retransplante, portadores de obesidade grau 3 (IMC maior do que 40,0 Kg/m2) e óbito.

Os pacientes incluídos no estudo serão avaliados após 24 horas de extubação orotraqueal, com
reavaliações diárias durante todo o período de internação na UTI até a alta da UTI para a enfermaria, onde será realizada uma última avaliação. Durante o estudo serão utilizados os seguintes recursos: eletromiografia de superfície (EMGs), dispositivo eletrônico de treinamento muscular inspiratório Powerbreathe® e Manovacuômetro digital.Previamente à avaliação muscular

diafragmática, serão registrados em ficha de avaliação informações relacionados ao paciente, procedimento cirúrgico e relacionadas à internação na UTI.

Para registros da atividade elétrica muscular diafragmática, será utilizado o aparelho de eletromiografia de superfície da marca EMG System do Brasil (modelo SA1000V3) que estaráconectado através de software próprio instalado em um notebook da marca DELL, modelo InterPentiun. A

aquisição dos sinais serão feitas em uma frequência de 500Hz e a sensibilidade de amplitude do sinal de 500 VA. Para sua realização o paciente deverá está posicionado em decúbito dorsal, com cabeceira elevada a 35 graus. Serão posicionados quatro eletrodos bipolares auto-adesivos da marca 3M do Brasil®, sendo dois na região paraxifóide, a 5cm de distância, dois na região da margem costal bilateral, à uma margem de 16cm de distância. Além disso, serão posicionados outros dois eletrodos na mão, sendo um na região anterior e outro na região posterior, sendo estes, eletrodos de referência. O paciente será guiado através de comandos verbais padronizados para que realize inspirações e expirações profundas, durante 10 segundos para obtenção da atividade elétrica muscular, que será processada e plotada, no qual serão analisados posteriormente os valores de RMS (Root Mean Square). Em seguida à EMGs, será realizada a avaliação dinâmica da força muscular inspiratória, utilizando o dispositivo da marca Powerbreathe®, modelo KH2 (Powerbreathe®, IMT Technologies Ltd., Birmingham, Inglaterra). Mantendo o mesmo posicionamento, em decúbito dorsal, no entanto, com a cabeceira elevada a 40º. Será adaptado o bocal do equipamento entre os lábios do paciente e um clipe nasal. Assim, o paciente será encorajado a realizar 10 inspirações profundas em sequência (sem descanso entre as tentativas) em uma válvula com passagem livre de ar, para que o dispositivo seja capaz de registrar a variação do fluxo inspiratório e calcular o valor (em cmH20) correspondente à força muscular inspiratória dinâmica, que será plotada no software Breathlink®, com registros dos dados em uma taxa de amostragem de 500Hz. Após plotagem no software, será analisado o valor correspondente ao S-Index. Ao final da avaliação será realizada medidas de PImax a pressão inspiratória máxima), através de manovacuômetro digital, portátil da marca MVD 300 Globalmed®. O paciente permanecerá no mesmo posicionamento e será adaptado o bocal do equipamento entre os lábios do paciente e um clipe nasal. Em seguida, o paciente será instruído sobre como realizar os esforços inspiratórios, que serão precedidos de uma exalação profunda, no nível do volume residual. E então, receberá incentivos para a realização de esforços inspiratórios máximos, cerca de três vezes, com intervalos de 30 segundos entre eles.Será considerado o maior

valor de PImáx obtido e registrado em ficha de avaliação. Critério de Inclusão: Pacientes acima de 18 anos, de ambos os sexos, submetidos a cirurgia de transplante hepático, e que estão extubados a um período mínimo de vinte e quatro horas, onde deverão estar conscientes e orientados para poderem obedecer aos comandos realizados pela equipe da fisioterapia. Critério de Exclusão:pacientes que por algum motivo forem re-intubados, ou seja, necessitarem voltar à ventilação mecânica, que não esteja consciente e orientado o suficiente para obedecerem aos comandos e re-transplantes hepáticos.

Objetivo da Pesquisa:

Avaliar a performance da musculatura inspiratória de pacientes no pós-operatório de transplante hepático,através da eletromiografia de superfície (EMGs), avaliação da força estática e dinâmica através da PImáx e S-Index de Powerbreathe®, respectivamente.Objetivos específicos: 1-Verificar se existe diferença nos valores de RMS (Root Mean Square) de EMGs do músculo diafragma durante a estadia do paciente na UTI.2-Verificar alterações da força dinâmica dos músculos inspiratórios, através dos valores de S-Index por meio do dispositivo Powerbreathe® durante internação na UTI.3-Analisar se existe alteração nos valores de PImáx durante internação dos pacientes submetidos ao TxH. 4-Correlacionar valores de RMS, de PImáx e S -Index com o tempo de ventilação mecânica e falha de
extubação orotraqueal.

Avaliação dos Riscos e Benefícios:

Riscos: Os riscos da intervenção poderão ser: fadiga muscular durante as solicitação de inspirações e expirações na aplicação dos procedimentos,desconforto de alguma forma relacionada aos eletrodos autoadesivos que serão colocados na pele e dor devido a incisão cirúrgica.

Benefícios:Essa pesquisa ira trazer informações importantes sobre a evolução do músculo diafragma após a realização do transplante hepático, levando a repercussões positivas tanto no período da internação quanto pós-operatório, e otimizar a melhor qualidade de atendimento e intervenção precoce na fraqueza muscular diafragmática neste paciente caso necessário. Não haverá benefícios diretos para os participantes da pesquisa.

Comentários e Considerações sobre a Pesquisa:

Trata-se de um estudo para conclusão de curso onde as fisioterapeutas Gabriela Martins Flauzino,Isabela Vitalis Stripolli, serão orientados pelos fisioterapeutas Rodrigo Marques Tonella e Rayssa Pistilli Duarte. Serão avaliados 15 participantes que realizaram transplante hepático que realizarão

Endereço: Rua Tessália Vieira de Camargo, 126
Bairro: Barão Geraldo **CEP:** 13.083-887
UF: SP **Município:** CAMPINAS
Telefone: (19)3521-8936 **Fax:** (19)3521-7187 **E-mail:** cep@fcm.unicamp.br

avaliação da musculatura diafragmática após no mínimo 24 horas da extubação traqueal e a cada 24 horas até a alta da UTI para a enfermaria, onde será realizada uma última avaliação. Durante o estudo serão utilizados os seguintes recursos: eletromiografia de superfície (EMGs), dispositivo eletrônico de treinamento muscular inspiratório Powerbreathe® e Manovacuômetro digital.Os procedimentos são não dolorosos e não invasivos, no entanto, podem causar algum desconforto pois participantes estão em pós-operatório.

Considerações sobre os Termos de apresentação obrigatória:

Foram avaliados os seguintes documentos: 1-Folha de rosto "Doc2.pdf" que se encontra preenchida e assinada pelo Coordenador de Assistência do HC/UNICAMP . 2 - "PB_INFORMAÇÕES_BÁSICAS_DO_PROJETO_1415477.pdf" e "PROJETO_corrigido.docx" que dão o detalhamento da metodologia do projeto. 3-"TCLE_CORRIGIDO.docx" que apresenta o TCLE que está redigido seguindo os moldes preconizados pelo CEP/UNICAMP e atende aos requisitos da resolução 466/12. Foram feitas algumas modificações, mas faltou a correção do item benefícios.4- "carta_resposta.pdf" que descreve as modificações feitas no projeto e TCLE para atender ao Parecer do CEP.5- No documento "autorizacao_coleta.pdf" o responsável pela Unidade de Terapia Intensiva de Transplantes dá o anuência para o projeto a ser realizado na Unidade.Foram feitas algumas modificações, mas faltou a correção do item benefícios. As última versão do TCLE "TCLE_JANEIRO.docx" e "PB_INFORMAÇÕES_BÁSICAS_DO_PROJETO_1415477.pdf" com a respectiva "CARTA_RESPOSTA_2020.docx" foram anexadas à plataforma Brasil para atender parecer 3.759.558 do CEP UNICAMP.

Conclusões ou Pendências e Lista de Inadequações:

Aprovado.

Considerações Finais a critério do CEP:

- O participante da pesquisa deve receber uma via do Termo de Consentimento Livre e Esclarecido, na íntegra, por ele assinado (quando aplicável).

- O participante da pesquisa tem a liberdade de recusar-se a participar ou de retirar seu consentimento em qualquer fase da pesquisa, sem penalização alguma e sem prejuízo ao seu cuidado (quando aplicável).

- O pesquisador deve desenvolver a pesquisa conforme delineada no protocolo aprovado. Se o pesquisador considerar a descontinuação do estudo, esta deve ser justificada e somente ser

Endereço: Rua Tessália Vieira de Camargo, 126
Bairro: Barão Geraldo **CEP:** 13.083-887
UF: SP **Município:** CAMPINAS
Telefone: (19)3521-8936 **Fax:** (19)3521-7187 **E-mail:** cep@fcm.unicamp.br

realizada após análise das razões da descontinuidade pelo CEP que o aprovou. O pesquisador deve aguardar o parecer do CEP quanto à descontinuação, exceto quando perceber risco ou dano não previsto ao participante ou quando constatar a superioridade de uma estratégia diagnóstica ou terapêutica oferecida a um dos grupos da pesquisa, isto é, somente em caso de necessidade de ação imediata com intuito de proteger os participantes.

- O CEP deve ser informado de todos os efeitos adversos ou fatos relevantes que alterem o curso normal do estudo. É papel do pesquisador assegurar medidas imediatas adequadas frente a evento adverso grave ocorrido (mesmo que tenha sido em outro centro) e enviar notificação ao CEP e à Agência Nacional de Vigilância Sanitária – ANVISA – junto com seu posicionamento.

- Eventuais modificações ou emendas ao protocolo devem ser apresentadas ao CEP de forma clara e sucinta, identificando a parte do protocolo a ser modificada e suas justificativas e aguardando a aprovação do CEP para continuidade da pesquisa. Em caso de projetos do Grupo I ou II apresentados anteriormente à ANVISA, o pesquisador ou patrocinador deve enviá-las também à mesma, junto com o parecer aprovatório do CEP, para serem juntadas ao protocolo inicial.

- Relatórios parciais e final devem ser apresentados ao CEP, inicialmente seis meses após a data deste parecer de aprovação e ao término do estudo.

-Lembramos que segundo a Resolução 466/2012 , item XI.2 letra e, “cabe ao pesquisador apresentar dados solicitados pelo CEP ou pela CONEP a qualquer momento”.

-O pesquisador deve manter os dados da pesquisa em arquivo, físico ou digital, sob sua guarda e responsabilidade, por um período de 5 anos após o término da pesquisa.

Este parecer foi elaborado baseado nos documentos abaixo relacionados:

Tipo Documento	Arquivo	Postagem	Autor	Situação
Informações Básicas do Projeto	PB_INFORMAÇÕES_BÁSICAS_DO_PROJETO_1415477.pdf	07/01/2020 17:03:57		Aceito
Outros	CARTA_RESPOSTA_2020.docx	07/01/2020 17:03:35	Rayssa Pistilli Duarte	Aceito

TCLE / Termos de Assentimento / Justificativa de Ausência	TCLE_JANEIRO.docx	07/01/2020 17:03:22	Rayssa Pistilli Duarte	Aceito
Outros	carta_resposta.pdf	05/12/2019 22:38:02	Rayssa Pistilli Duarte	Aceito
Outros	gabriela.pdf	11/11/2019 21:54:39	Rayssa Pistilli Duarte	Aceito
Declaração de Pesquisadores	isabela.pdf	11/11/2019 21:54:27	Rayssa Pistilli Duarte	Aceito
Outros	autorizacao_coleta.pdf	11/11/2019 21:52:56	Rayssa Pistilli Duarte	Aceito
Projeto Detalhado / Brochura Investigador	PROJETO_corrigido.docx	11/11/2019 21:49:16	Rayssa Pistilli Duarte	Aceito
TCLE / Termos de Assentimento / Justificativa de Ausência	TCLE_CORRIGIDO.docx	11/11/2019 21:49:03	Rayssa Pistilli Duarte	Aceito
Outros	identidade.jpg	23/09/2019 15:11:14	Rayssa Pistilli Duarte	Aceito
Folha de Rosto	Doc2.pdf	16/09/2019 19:26:59	Rayssa Pistilli Duarte	Aceito

Situação do Parecer:
Aprovado

Necessita Apreciação da CONEP:
Não

CAMPINAS, 17 de Janeiro de 2020

Assinado por:
Renata Maria dos Santos Celeghini
(Coordenador(a))

Endereço: Rua Tessália Vieira de Camargo, 126
Bairro: Barão Geraldo **CEP:** 13.083-887
UF: SP **Município:** CAMPINAS
Telefone: (19)3521-8936 **Fax:** (19)3521-7187 **E-mail:** cep@fcm.unicamp.br
