# Supplementary material for: Inspiratory muscle strength and post-liver transplantation outcomes: safety of measurement in the ICU ‒ an observational study
Source: Clinics (Sao Paulo). 2026 Sep 22;81:101079. doi: 10.1016/j.clinsp.2026.101079 (PMC13629311; doi:10.1016/j.clinsp.2026.101079)
Supplement: Supplementary file 2 [file mmc3.pdf]

Ensaio clínico RBR-3v4k4d6 aprovado Caixa de entrada ×

ReBEC <contato@rebecmsg.com.br>
para mim, ReBEC

Esta é uma mensagem automática. Por favor não responda.

Prezado Registrante,

Temos o prazer de informar que seu estudo foi publicado no Registro Brasileiro de Ensaios Clínicos (ReBEC) com o número RBR-3v4k4d6 . Agradecemos por seu registro e colaboração e, desde já, nos colocamos à disposição para esclarecer quaisquer dúvidas que possam surgir, seja em caso de atualização do registro ou, até mesmo, uma nova submissão. Por favor, não hesite em contactar-nos. Cordialmente, ReBEC Staff - ReBEC/ICICT/LIS Av. Brasil 4036 - Maré - sala 807 Rio de Janeiro RJ CEP: 21040-360 Tel: +55(21)3882-9227

Acesso: https://ensaiosclinicos.gov.br/rg/RBR-3v4k4d6

Esta é uma mensagem automática. Por favor não responda.
