# Supplementary material for: Inspiratory muscle strength and post-liver transplantation outcomes: safety of measurement in the ICU ‒ an observational study
Source: Clinics (Sao Paulo). 2026 Sep 22;81:101079. doi: 10.1016/j.clinsp.2026.101079 (PMC13629311; doi:10.1016/j.clinsp.2026.101079)
Supplement: Supplementary file 3 [file mmc4.pdf]

Consultas

ANVISA - Agência Nacional de Vigilância Sanitária

Detalhes do Produto	
**Nome da Empresa Detentora da Notificação ou do Registro do Dispositivo Médico**	HOMED EQUIPAMENTOS MEDICO HOSPITALAR LTDA - EPP
**CNPJ do Detentor da Notificação ou do Registro do Dispositivo Médico**	64.835.721/0001-02
**Autorização de Funcionamento da Empresa**	1.03.200-9
**Nome do Dispositivo Médico**	MANOVACUÔMETRO DIGITAL HOMED
**Nome Técnico do Dispositivo Médico**	Aparelho Para Fisioterapia
**Número da Notificação ou do Registro do Dispositivo Médico**	10320099006
**Situação da Notificação ou do Registro do Dispositivo Médico**	Válido
**Processo da Notificação ou Registro do Dispositivo Médico**	25351410696202413
**Fabricante Legal do Dispositivo Médico**	FABRICANTE: HOMED EQUIPAMENTOS MEDICO HOSPITALAR LTDA - EPP - BRASIL - CNPJ / Código Único: 64835721000102 - Endereço: RUA GASTÃO DA CUNHA 125 VILA PAULISTA 04361090
**Classificação de Risco do Dispositivo Médico**	II - MEDIO RISCO
**Data de Início da Vigência da Notificação ou do Registro do Dispositivo Médico**	21/10/2024
**Data de Vencimento da Notificação ou do Registro do Dispositivo Médico**	VIGENTE

Tipo de Arquivo	Arquivos	Expediente, data e hora de inclusão
INSTRUÇÕES DE USO OU MANUAL DO USUÁRIO DO PRODUTO	MVD300U_MVD300 - Manual do Usuário - Rev 8.0a.pdf	1487390246 - 29/10/2024 15:49:42

Modelo Produto Médico
MVD300-U: MANOVACUÔMETRO DIGITAL HOMED USB
MVD300: MANOVACUÔMETRO DIGITAL HOMED
