# Supplementary material for: Inspiratory muscle strength and post-liver transplantation outcomes: safety of measurement in the ICU ‒ an observational study
Source: Clinics (Sao Paulo). 2026 Sep 22;81:101079. doi: 10.1016/j.clinsp.2026.101079 (PMC13629311; doi:10.1016/j.clinsp.2026.101079)
Supplement: Supplementary file 4 [file mmc5.pdf]

TREINAMENTO MUSCULAR RESPIRATÓRIO COM POWERBREATHE®

Pacientes elegíveis para treinamento muscular respiratório (TMR)

- Adultos ou crianças > 7 anos sob supervisão.
- Desmame difícil da ventilação mecânica.
- Dispneia para atividades funcionais.
- PiMáx < - 30 cmH2O.
- MRC < 48.
- Fração de espessamento diafragmático < 20%.

Contra indicações e Precauções

- Histórico de pneumotórax espontâneo.
- Hipertensão pulmonar.
- Enfisema pulmonar.
- Osteoporose acentuada com histórico de fraturas de costelas.
- Dessaturação durante o uso de TMI (SpO2 < 88% para pacientes pneumopatas e SpO2<92% para pacientes sem diagnóstico de pneumopatia).
- Asma em pacientes com baixa percepção de sintomas, com agravamento severo.
- Perfuração do tímpano.
- Pacientes com volume e pressão diastólica final do ventrículo esquerdo elevado.
- Pacientes com sinais e sintomas de piora na insuficiência cardíaca após TMR / TMI.

Avaliação da musculatura respiratória

- Manovacuometria com mensuração da pressão inspiratória máxima (PiMáx).
- Para pacientes com via aérea artificial: MIP disponível nos equipamentos de ventilação mecânica invasiva.
- Ultrassonografia - avaliação da fração de espessamento diafragmático.

Powerbreathe Classic ® e Medic Plus ®

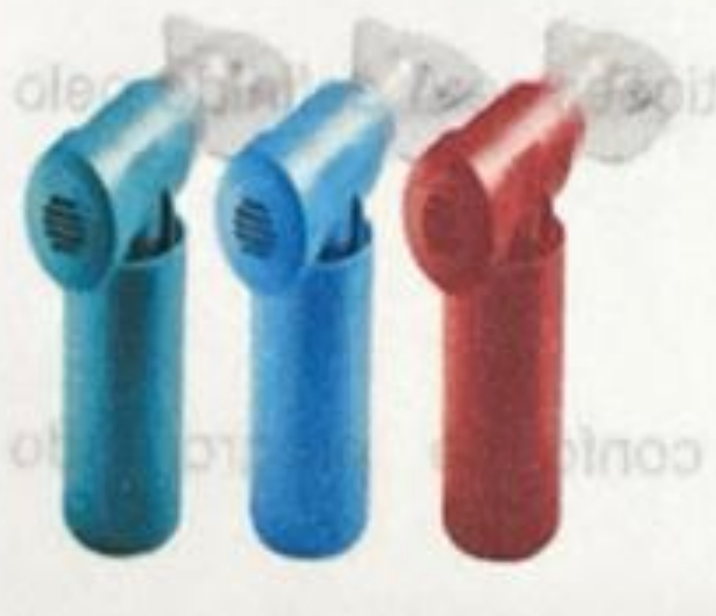

Ajuste da carga	Carga (cmH_2O) 1	2	3	4	5	6	7	8	9
Resistência Leve - COR VERDE e COR BRANCA	10	20	30	40	50	60	70	80	90
Resistência Moderada - COR AZUL	10	30	50	70	90	110	130	150	170
Resistência Pesada - COR VERMELHA	10	40	70	100	130	160	190	220	250

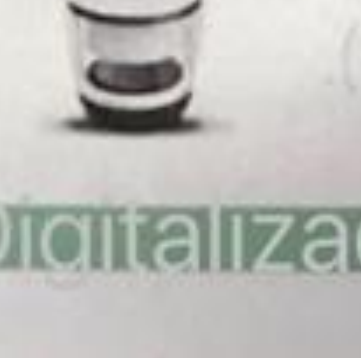

Ajuste da carga	Carga (cmH_2O) 0	1	2	3	4	5	6	7	8	9	10
MEDIC PLUS	9	16	23	29	36	43	50	57	64	71	78

Digitalizada com CamScanner

TREINAMENTO MUSCULAR RESPIRATÓRIO COM POWERBREATHE®

Powerbreathe KH2 ®

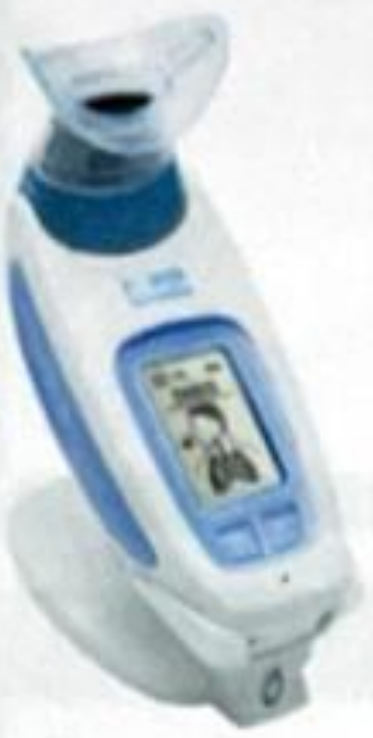

- Carga de treinamento variável manualmente ajustável (3-200 cmH_2O).
- Carga de treinamento eletrônico variável.
- Medição de Pressão Inspiratória Máxima (MIP), Pico de Fluxo Inspiratório (PIF) e S-index.
- Resultados de treinamento (volume inalado, tensão, carga e intensidade de treinamento).
- Orientação de ritmo de respiração (bip sonoro).

Critérios para prescrição e ajuste do treinamento

- **Ajuste da carga inicial de treinamento:**
  - **Treino de força:** utilizar a carga equivalente a 50% da PiMáx e solicitar 3 séries de 6 a 8 ciclos respiratórios, com intervalo de 1 a 2 minutos entre cada série.
  - **Treino de endurance:** utilizar a carga equivalente a 30 a 50% da PiMáx e solicitar 3 séries de 10 ciclos respiratórios, com intervalo de 1 a 2 minutos entre cada série.
  - O treinamento é individualizado. Pode-se modificar o número de séries, repetições e intervalo entre as séries.
  - **Atenção:** no intervalo entre as séries, se o paciente estiver em uso de oxigenoterapia ou ventilação mecânica invasiva, deve-se reconectar o dispositivo ou equipamento.

- **Intensidade:**
  - Escala de Borg modificada (escala de zero a dez): níveis de esforço entre 3 e 4.

- **Duração:**
  - O tempo da terapia e a quantidade de séries e repetições será definido pelo fisioterapeuta.

- **Frequência:**
  - O TMR deve ser aplicado pelo fisioterapeuta conforme programado inicialmente.

- **Progressão:**
  - Para progressão do treinamento deve-se tentar aumentar 10% da carga diariamente.

Obs: em pacientes internados, é recomendado associar o TMR a mobilização precoce e ao desmame ventilatório (se o paciente necessitar de VMI).

Digitalizada com CamScanner
