# Supplementary material for: Inspiratory muscle strength and post-liver transplantation outcomes: safety of measurement in the ICU ‒ an observational study
Source: Clinics (Sao Paulo). 2026 Sep 22;81:101079. doi: 10.1016/j.clinsp.2026.101079 (PMC13629311; doi:10.1016/j.clinsp.2026.101079)
Supplement: Supplementary file 5 [file mmc6.pdf]

PRESSÃO INSPIRATÓRIA MÁXIMA (PiMáx) e PRESSÃO EXPIRATÓRIA MÁXIMA (PeMáx)

Indicações

- Avaliar se há disfunção dos músculos ventilatórios.
- Avaliar a função dos músculos respiratórios em condições pré e P.O ou em pacientes com doenças neuromusculares.
- Avaliar a função dos músculos respiratórios para desmame da V.M.
- Avaliar a resposta da reabilitação pulmonar e treinamento muscular respiratório.
- Avaliar a habilidade de tossir e expectorar em pacientes com doenças neuromusculares.

Contra Indicações

Absolutas:

- IAM ou Angina Instável.
- HAS grave e descontrolada.
- Aneurisma de aorta.
- Pneumotórax não drenado.
- Fístulas pleurais.
- Cirurgias e traumas de V.A.S, tórax e abdômen.
- Hérnias abdominais.
- Hidrocefalia.
- Doenças com contra indicação para manobra de valsalva.

Relativas:

- Alterações de N.C que impeçam a compreensão e a execução do teste em pacientes sem via aérea artificial. Nestes casos é necessário realizar a mensuração com válvula unidirecional.
- Paralisia facial.

Riscos:

- Queda da saturação durante a realização do teste.
- Instabilidade hemodinâmica.
- Arritmia cardíaca.
- Hipertensão arterial.

Como realizar a medida em pacientes em Respiração Espontânea?

- **Materiais necessários:** manovacuômetro analógico ou digital, mangueira de silicone, conector com orifício de 2mm, filtro HMEF, clipe nasal e máscara ou bucal.
- **Antes da manobra:** adaptar o clipe nasal no paciente e fornecer o bucal para mensuração. Pode-se utilizar a máscara facial com correta vedação em pacientes que não conseguem realizar a preensão labial correta.
- **Manovacuômetro digital:** montar previamente, ligar o aparelho, selecionar a medida que irá realizar (PI ou PE) e clicar em início. Após o término, clicar em pausa e o visor mostrará as maiores medidas obtidas. O fisioterapeuta pode fornecer estímulo verbal através de frases durante o teste.
- **Para PiMáx:** solicitar ao paciente que expire todo o ar contido nos pulmões até o Volume Residual (VR), seguida de uma manobra de inspiração máxima sustentada pela boca.
- **Para PeMáx**: solicitar ao paciente que inspire de forma máxima, até atingir a Capacidade Pulmonar Total (CPT) seguida de uma expiração máxima sustentada. Nesta medida é recomendado a contenção da musculatura perioral para evitar a interferência destes músculos, bem como do mecanismo de fechamento glótico.
- É recomendado realizar 5 manobras, com duração de 2 segundos cada e repouso de 60 seg. entre as manobras. Após a realização, selecione 3 manobras aceitáveis. O ideal é que os valores das manobras não tenham diferenças maiores do que 10% entre elas.

CS Digitalizado com CamScanner

PRESSÃO INSPIRATÓRIA MÁXIMA (PiMáx) e PRESSÃO EXPIRATÓRIA MÁXIMA (PeMáx)

Materiais necessários - pacientes com via aérea artificial:

Para realizar a medida em paciente com via aérea artificial (tubo orotraqueal ou traqueostomia) será necessário: manovacuômetro analógico ou digital, válvula unidirecional (inspiratória e/ou expiratória), mangueira de silicone, conector branco, filtro HMEF.

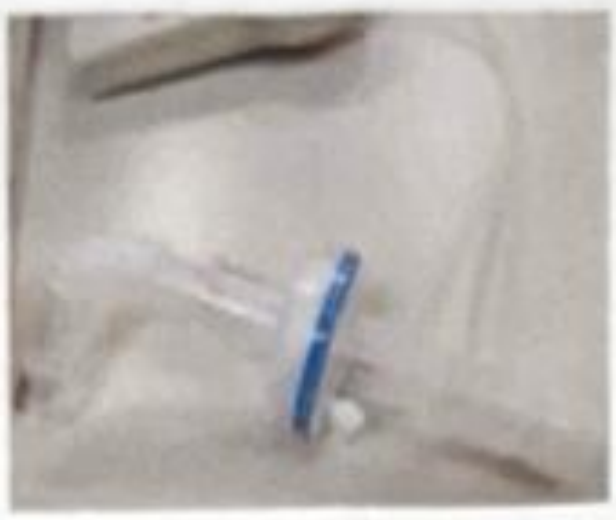

PiMáx

Montagem correta com mangueira de silicone, filtro HMEF, conector branco e válvula unidirecional para PiMáx

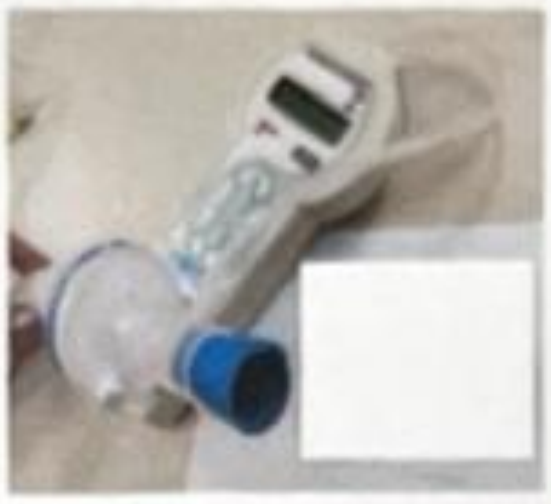

PeMáx

Montagem correta com mangueira de silicone, filtro HMEF, conector e válvula unidirecional para PeMáx

Como realizar a medida em pacientes com via aérea artificial

- Condições mínimas para o teste: paciente sem sedação, com estabilidade hemodinâmica e parâmetros ventilatórios na modalidade PSV com $FiO_2 \leq 40\%$ e PEEP ≤ 8 cmH_2O.
- Como sugestão, pode-se realizar a pré-oxigenação antes do teste, durante 2 minutos, para diminuir o risco de dessaturação durante o procedimento.
- Desconectar o paciente da via aérea artificial e conectar rapidamente o manovacuômetro, já previamente montado e ligado.
- Mensurar durante 40 segundos. Se o paciente estiver consciente, o fisioterapeuta pode fornecer estímulo verbal positivo através de frases.
- Clicar em pausa no manovacuômetro, reconectar o paciente a V.M e anotar a maior medida fornecida.
- Para PiMáx: solicitar ao paciente que expire todo o ar contido nos pulmões até o Volume Residual (VR), seguida de uma manobra de inspiração máxima sustentada pela boca. Quando a válvula unidirecional é utilizada, esta permite a expiração, enquanto a inspiração é bloqueada. O bloqueio da entrada de ar obriga o centro respiratório a estimular os músculos a realizar esforços maiores a cada ciclo respiratório. O paciente irá realizar sucessivos e progressivos esforços até a pressão na via aérea se tornar a mais negativa possível.
- Para PeMáx: solicitar ao paciente que inspire de forma máxima, até atingir a Capacidade Pulmonar Total (CPT) seguida de uma expiração máxima sustentada. Quando a válvula unidirecional é utilizada, esta permite a inspiração, enquanto a expiração é bloqueada. O bloqueio da entrada de ar obriga o centro respiratório a estimular os músculos a realizar esforços maiores a cada ciclo respiratório. O paciente irá realizar sucessivos e progressivos esforços até a pressão na via aérea se tornar a mais positiva possível.
- O repouso entre as 3 medidas deve ser de 60 segundos.
- Repetir a manobrar 3 vezes e anotar o valor mais alto de pressão alcançada.

LEMBRETES

- Se o paciente apresentar instabilidade, interromper o teste imediatamente.
- Se o paciente estiver em isolamento de contato, proteger o equipamento em saco plástico.

Sempre utilize o filtro HMEF para as medidas ventilatórias.

Digitalizada com CamScanner
