# Supplementary material for: Inspiratory muscle strength and post-liver transplantation outcomes: safety of measurement in the ICU ‒ an observational study
Source: Clinics (Sao Paulo). 2026 Sep 22;81:101079. doi: 10.1016/j.clinsp.2026.101079 (PMC13629311; doi:10.1016/j.clinsp.2026.101079)
Supplement: Supplementary file 6 [file mmc7.pdf]

Fisioterapia no Pós Operatório de Esofagectomia e Cirurgias Abdominais

Indicação:

- Pacientes submetidos a cirurgias abdominais e esofagectomias.

Pacientes candidatos a cirurgias abdominais podem apresentar descondicionamento físico, sarcopenia, fragilidade física, desnutrição, sedentarismo, inflamação sistêmica e em casos de transplante hepático, também pode ocorrer hiperamonemia e disfunção endócrina.

A redução do estado funcional pré-cirúrgico pode ocasionar em atrasos na alta hospitalar e prejudicar a qualidade de vida do paciente. A reabilitação pós-operatória pode auxiliar na redução das complicações cirúrgicas e na diminuição do tempo de internação hospitalar.

Admissão:

Os pacientes submetidos às cirurgias abdominais geralmente são extubados na recuperação pós anestésica e encaminhados em oxigenoterapia para UTI.

Comumente, os pacientes que realizaram Tx hepático são encaminhados para UTI sob efeito anestésico e em VMI.

Na admissão do paciente em VMI: priorizar o desmame da ventilação mecânica e extubação precoce (até 6 horas após admissão na UTI, quando possível).

Fisioterapia Respiratória:

Em cirurgias abdominais altas, as complicações pulmonares no pós-operatório (CPP) ocorrem com maior frequência.

- **Exercícios respiratórios:**
  - Exercícios respiratórios tanto para reexpansão pulmonar quanto para higiene brônquica.
  - Pacientes com secreção pulmonar: Huffing, drenagem autógena, oscilador oral de alta frequência, além da discussão com a equipe sobre a administração de analgesia quando indicado, devido a dor na incisão cirúrgica.
  - Atenção: pode ocorrer sangramentos nasais ou orais em pacientes no P.O Tx hepático. Se for necessário a aspiração durante a higiene brônquica deve ser realizado uma avaliação prévia dos exames laboratoriais (plaquetas, Hb e RNI) com a equipe. Alguns cuidados devem ser tomados, como: tamanho da sonda, posicionamento, avaliação da mucosa para identificação de lesões prévias, uso de cânula de guedel em aspiração oral e uso de lidocaína gel em aspiração nasal.
- **Treinamento Muscular Respiratório (TMR):**
  - Pacientes submetidos a cirurgia abdominal apresentam diminuição da PiMáx e redução da mobilidade diafragmática.
  - Como recomendação, mensurar a PiMáx e iniciar o TMR com Powerbreathe®, utilizando de 30 a 50% deste valor e 3 séries de 6 a 8 repetições.
- **Oxigenoterapia:**
  - Indicação: pacientes hipoxêmicos, utilizar com dispositivo + adequado para SpO2 alvo.
- **VMNI:**
  - Indicação: pacientes com riscos de falha de extubação, como terapia de resgate em IRpA ou associada ao exercício.
    - Em pacientes submetidos a esofagectomia e gastrectomias, a utilização da VMNI deve ser avaliada com cautela pela equipe multidisciplinar e cirúrgica. Se a equipe consentir o uso da VMNI, deve-se atentar para adaptar com níveis pressóricos baixos e evitar pressurização acima de 20 cmH2O.
  - Contra indicação: medida preventiva de complicações pulmonares.
  - Atenção ao risco de broncoaspiração em pacientes que apresentam distensão abdominal, náuseas e vômitos, incapacidade de mobilizar secreções ou aerofagia.
- **CNAF:**
  - Indicação: pacientes com insuficiência respiratória hipoxêmica.
  - Contra indicação: medida preventiva de complicações pulmonares.

Digitalizada com CamScanner

Contra indicações:

- Instabilidade hemodinâmica:
  - Hipertensão arterial não controlada (PAS > 180 e PAD > 110 mmHg)
  - Hipotensão arterial (PAM < 65 mmHg)
  - Hipotensão ortostática sintomática com queda > 20 mmHg
  - Frequência cardíaca (FC) ≤ 50 ou ≥ 130 bpm
  - Arritmias ventriculares complexas ou sintomáticas
- Estado febril ou sinais de infecção sistêmica
- Outros fatores limitantes (encefalopatia hepática, osteomusculares, psíquicos, etc.)
- Evento neurológico isquêmico ou hemorrágico agudo ou não-controlado
- Hipertensão intracraniana
- Frequência respiratória (FR) ≤ 12 ou ≥ 40 rpm
- Fração inspirada de oxigênio (FiO_2) ≥ 60 % e PEEP ≥ 10 cmH_2O
- Alterações metabólicas como hipocalemia, hipercalcemia, hipercalemia ou hipovolemia
- Sangramento ativo
- RNI > 3
- Hemoglobina < 7 g/dL
- Plaquetas < 20 000 células / m3

Para maiores informações sobre as contra-indicações e critérios de segurança para mobilização precoce, acesse o POP de Mobilização Precoce.

Protocolo de mobilização

Na avaliação fisioterapêutica, a condição de mobilidade do paciente internado na UTI também é direcionada pela **Perme Escore.**

A prescrição de exercício deve ser individualizada, com atenção às condições clínicas, hemodinâmicas, dia do pós operatório, limitações individuais e comorbidades dos pacientes.

As atividades devem ser realizadas com o paciente sob monitorização. Em casos de mobilização fora do leito, também é recomendado a adaptação prévia da cinta abdominal, que garante conforto e segurança para o paciente durante a execução da mobilização.

DIA DE P.O.	INTERVENÇÃO
P.O. Imediato	• Exercícios ativos globais com o paciente no leito, conforme colaboração, dos seguintes grupos musculares:<br>◦ Quadríceps, abdutores, adutores e panturrilhas<br>◦ Bíceps braquial, deltóide e tríceps.<br>• Orientações quanto à mobilidade no leito.
1º P.O.	• Exercícios ativos globais com o paciente no leito:<br>◦ Quadríceps, abdutores, adutores e panturrilhas<br>◦ Bíceps braquial, deltóide e tríceps.<br>• Sedestação à beira do leito e / ou na poltrona.
2º P.O.	• Exercícios do 1º P.O. com resistência (halter, faixa elástica, tornozeleiras) + marcha estacionária, pode ser substituída pelo cicloergômetro.<br>• Sedestação na poltrona.<br>• Deambulação: mínimo de 5 metros.
3º P.O.	• Exercícios do 2º P.O.<br>• Deambulação 15 metros.<br>• Sedestação na poltrona.
4º P.O.	• Deambulação > 30 metros.<br>• Sedestação na poltrona.<br>• Avaliação quanto a liberação de deambulação do paciente com o acompanhante.
5º P.O. até alta hospitalar	• Deambulação > 30 metros.<br>• Subir e descer 15 degraus de escada.<br>• Sedestação em poltrona.

CS Digitalizada com CamScanner

Critérios para Prescrição e Avaliação do Exercício:

- **Intensidade:**
  - Borg modificada: entre 3 e 4.
  - FC limitada em 30 bpm acima da FC de repouso. Atenção: pode sofrer influência da utilização de DVA e betabloqueadores.
- **Duração:**
  - O tempo da terapia será definido pelo fisioterapeuta.
  - Período de repouso entre as séries: 1 a 2 min ou de acordo com o paciente.
- **Frequência:**
  - Mobilização: 1 a 2 vezes por dia, de acordo com a avaliação do fisioterapeuta.
- **Progressão:**
  - Aumentar inicialmente a duração de 10 a 15 minutos no tempo de exercício e após aumentar a intensidade.
  - Aumentar a distância de caminhada percorrida e incluir o treino de escadas.

CUIDADOS DURANTE A FISIOTERAPIA MOTORA	
**Avaliar resposta clínica e hemodinâmica**	• Manter paciente sob monitorização.<br>• Atentar à presença de arritmias.<br>• Monitorar pressão arterial pré e pós-exercício nas trocas posturais e em ortostatismo.<br>• Orientar o paciente para não realizar manobra de valsalva durante o exercício.
**Avaliar padrão respiratório e sinais de hipoxemia**	• Manter SpO_2 dentro do alvo individualizado.<br>• Se necessário, utilizar dispositivos para suplementação de oxigênio ou VMNI durante o exercício.
**Observar drenos e dispositivos (sondas, cateteres)**	• Inserção, função e débitos dos drenos.<br>• Individualizar a mobilização conforme quadro clínico.<br>• Observar possíveis sinais de deiscências da ferida operatória.

- **Sinais de intolerância ao esforço:**
  - Dor torácica.
  - Sensação de fadiga.
  - Dispneia e/ou Taquipneia acentuada.
  - Palidez e náusea (má perfusão tecidual ou débito cardíaco inadequado).
  - Oscilações pressóricas significativas (hipotensão ou HAS).
  - Hipotensão postural.
  - Taquicardia: FC > 30 bpm da FC de repouso.
  - Manifestação ou agravamento de arritmias.
  - Queda da saturação periférica de oxigênio.

Caso o paciente apresente algum dos sinais descritos acima, o protocolo deve ser interrompido até a resolução do quadro e comunicado imediatamente à equipe.

Atenção: Complicações cirúrgicas como necessidade de reabordagem cirúrgica, desmame difícil da VM e instabilidade hemodinâmica não seguem o protocolo padrão sugerido.

Transferência de cuidados para a unidade de internação de adultos (UIA):

No decorrer do P.O, com a melhora progressiva do paciente, a retirada dos dispositivos e a evolução favorável na reabilitação são realizados os ajustes finais do tratamento, bem como orientações gerais para preparar o paciente para a alta hospitalar e início do acompanhamento ambulatorial.

O acompanhamento fisioterapêutico é mantido até que o paciente alcance a meta terapêutica proposta, garantindo a continuidade do cuidado, reabilitação funcional e retorno precoce às atividades de vida diária.

Digitalizada com CamScanner
