# Supplementary material for: Inspiratory muscle strength and post-liver transplantation outcomes: safety of measurement in the ICU ‒ an observational study
Source: Clinics (Sao Paulo). 2026 Sep 22;81:101079. doi: 10.1016/j.clinsp.2026.101079 (PMC13629311; doi:10.1016/j.clinsp.2026.101079)
Supplement: Supplementary file 7 [file mmc1.docx]

**CREDIT AUTHOR STATEMENT**

**Rayssa Pistilli Duarte:** Conceptualization; Methodology, Validation; Investigation; Resources; Data Curation; Writing - Original Draft; Writing - Review & Editing; Visualization; Supervision; Project administration.

**Rodrigo Marques Tonella: Data Curation,** Writing - Review & Editing; Visualization; Supervision.

**Ligia dos Santos Roceto Ratti: Data Curation;** Writing - Review & Editing; Visualization; Supervision.

**Lilian Elisabete Bernardes Delazari:** Formal analysis.

**Gabriela Maria de Oliveira:** Investigation; Resources; Data Curation.

**Leticia de Souza Campos Assis:** Investigation; Resources; D.ata Curation

**Mariana Bueno da Rosa:** Investigation; Resources; Data Curation.

**Edmilson Florêncio Pinto Junior:** Investigation; Resources; Data Curation.

**Enrico de Francisco Magnani:** Formal analysis, Writing - Review & Editing.

**Ilka de Fátima Santana Ferreira Boin:** Methodology; Project administration; Visualization; Supervision.
